# COVID-19 Mechanisms in the Human Body—What We Know So Far

**DOI:** 10.3389/fimmu.2021.693938

**Published:** 2021-11-01

**Authors:** Ashutosh Kumar, Ravi K. Narayan, Pranav Prasoon, Chiman Kumari, Gurjot Kaur, Santosh Kumar, Maheswari Kulandhasamy, Kishore Sesham, Vikas Pareek, Muneeb A. Faiq, Sada N. Pandey, Himanshu N. Singh, Kamla Kant, Prakash S. Shekhawat, Khursheed Raza, Sujeet Kumar

**Affiliations:** ^1^ Etiologically Elusive Disorders Research Network (EEDRN), New Delhi, India; ^2^ Department of Anatomy, All India Institute of Medical Sciences (AIIMS), Patna, India; ^3^ Department of Anatomy, Andaman and Nicobar Islands Institute of Medical Sciences, Port Blair, India; ^4^ Pittsburgh Center for Pain Research, School of Medicine, University of Pittsburgh, Pittsburgh, PA, United States; ^5^ Department of Anatomy, Postgraduate Institute of Medical Education and Research (PGIMER), Chandigarh, India; ^6^ School of Pharmaceutical Sciences, Shoolini University, Solan, India; ^7^ Department of Anesthesiology and Critical Care Medicine, School of Medicine, Johns Hopkins University, Baltimore, MD, United States; ^8^ Department of Biochemistry, Maulana Azad Medical College (MAMC), New Delhi, India; ^9^ Department of Anatomy, All India Institute of Medical Sciences (AIIMS), Mangalagiri, Vijayawada, India; ^10^ Center for Cognitive and Brain Sciences, Indian Institute of Technology Gandhinagar, Gandhinagar, Gujarat, India; ^11^ New York University (NYU) Langone Health Center, NYU Robert I. Grossman School of Medicine, New York, NY, United States; ^12^ Department of Zoology, Banaras Hindu University (BHU), Varanasi, India; ^13^ Department of Systems Biology, Columbia University Irving Medical Center, New York, NY, United States; ^14^ Department of Microbiology, All India Institute of Medical Sciences (AIIMS), Bathinda, India; ^15^ Department of Clinical Hematology, National Institute of Medical Sciences, Jaipur, India; ^16^ Department of Anatomy, All India Institute of Medical Sciences (AIIMS), Deoghar, India; ^17^ Center for Proteomics and Drug Discovery, Amity Institute of Biotechnology, Amity University, Maharashtra, India

**Keywords:** COVID-19, SARS-CoV-2, pathogenesis, immune response, organotropism

## Abstract

More than one and a half years have elapsed since the commencement of the coronavirus disease 2019 (COVID-19) pandemic, and the world is struggling to contain it. Being caused by a previously unknown virus, in the initial period, there had been an extreme paucity of knowledge about the disease mechanisms, which hampered preventive and therapeutic measures against COVID-19. In an endeavor to understand the pathogenic mechanisms, extensive experimental studies have been conducted across the globe involving cell culture-based experiments, human tissue organoids, and animal models, targeted to various aspects of the disease, *viz.*, viral properties, tissue tropism and organ-specific pathogenesis, involvement of physiological systems, and the human immune response against the infection. The vastly accumulated scientific knowledge on all aspects of COVID-19 has currently changed the scenario from great despair to hope. Even though spectacular progress has been made in all of these aspects, multiple knowledge gaps are remaining that need to be addressed in future studies. Moreover, multiple severe acute respiratory syndrome coronavirus 2 (SARS-CoV-2) variants have emerged across the globe since the onset of the first COVID-19 wave, with seemingly greater transmissibility/virulence and immune escape capabilities than the wild-type strain. In this review, we narrate the progress made since the commencement of the pandemic regarding the knowledge on COVID-19 mechanisms in the human body, including virus–host interactions, pulmonary and other systemic manifestations, immunological dysregulations, complications, host-specific vulnerability, and long-term health consequences in the survivors. Additionally, we provide a brief review of the current evidence explaining molecular mechanisms imparting greater transmissibility and virulence and immune escape capabilities to the emerging SARS-CoV-2 variants.

## Introduction

The ongoing pandemic of coronavirus disease 2019 (COVID-19) has taken a heavy toll on human lives globally (~4.8 million until October 8, 2021, per WHO data). COVID-19 is caused by severe acute respiratory syndrome coronavirus 2 (SARS-CoV-2) (1), an enveloped positive-sense single-stranded RNA virus belonging to the genus betacoronaviruses (BCoVs). BCoVs have other members like SARS-CoV-1 and the Middle East respiratory syndrome coronavirus (MERS-CoV) that have caused the respiratory syndrome epidemic of SARS-2002/2003 and MERS-2012, respectively (1). The extreme paucity of knowledge, especially in the initial period, about the interaction of the new coronavirus strain, SARS-CoV-2, with the host greatly hampered the preventive and therapeutic management of the pandemic. However, the tremendous research done across the globe has resulted in rigorous scientific evidence on all the aspects of COVID-19 at an unprecedented speed and scale. The emerging facts about pathogenic mechanisms in COVID-19 have helped to put a break on the uncontrolled spread of the pandemic by the development of various preventive measures including effective vaccines and by improved therapeutic management. Even though currently there is no effective drug for COVID-19, the current status of the research is raising hope for the future. The emergence of multiple SARS-CoV-2 variants with greater transmissibility/virulence and immune escape capabilities is an unfortunate turn in the current course (2020–2021) of the pandemic. Evidence is emerging that can explain improved virus–host interactions and immune escape capabilities of the variants (2–4); however, whether the variants have altered tissue type/organ-specific pathogenesis is least understood. In this review, we precisely discuss the current evidence for the pathogenic mechanisms of COVID-19 in humans, including virus–host interactions, pulmonary and other systemic manifestations, immunological dysregulations, complications, host-specific vulnerability, and long-term health issues in survivors. The article is intended to impart a comprehensive understanding of the key pathogenic mechanisms driving clinical manifestations and patient outcomes in COVID-19 and highlight the knowledge gaps that may need further attention from the researchers. Additionally, we also discuss in brief the current evidence explaining molecular mechanisms imparting greater transmissibility and virulence and immune escape capabilities to the emerging SARS-CoV-2 variants.

## Symptomatology of COVID-19

**Figure 1:**
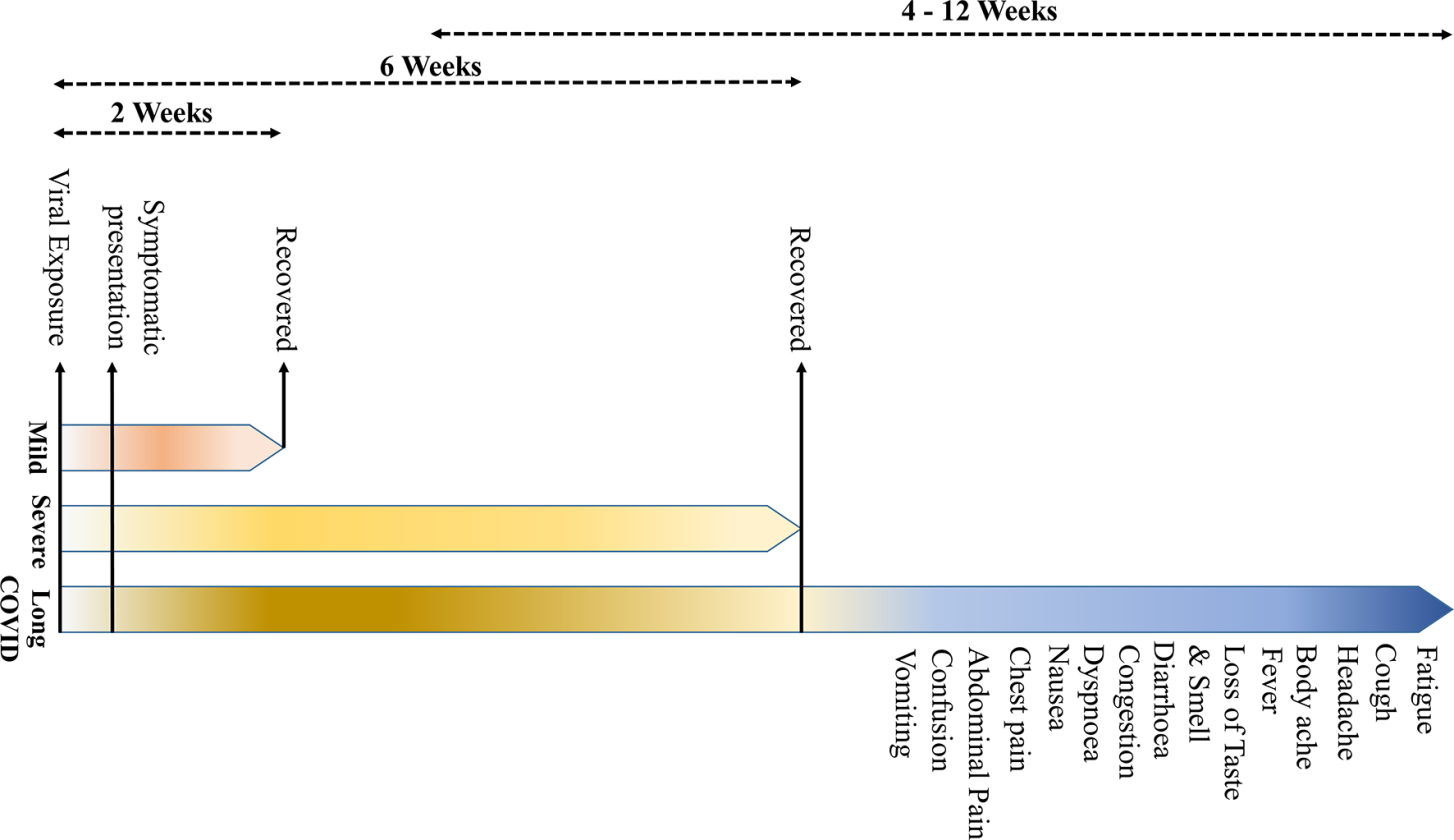
The symptomatology of coronavirus disease 2019 (COVID-19). (The onset of the clinical symptoms occurs in average 5–6 days after exposure, and normally, those with mild symptoms recover within 2 weeks; however, in severe cases, the recovery may extend up to 6 weeks. Persistence of the disease or, after complete recovery, emergence of new ailments, together known as “long COVID” may occur in some patients. The “long COVID” is chiefly characterized by the presence of fatigue, headache, dyspnea, and anosmia, which may persist for 4–12 weeks.

COVID-19 is primarily described as a disease-causing severe acute respiratory syndrome (SARS); however, the systemic manifestations involving other organs, including the central nervous system (CNS), are very usual (5) (**Table 1**). The onset of the symptoms occurs on average 5–6 days after exposure, and normally, those with mild symptoms recover within 2 weeks; however, in severe cases, the recovery may extend up to 6 weeks (**Figure 1**). Of note, in some patients, regardless of the disease severity, the symptoms may persist or recur for weeks or months following initial recovery (17). Persistence of the disease or after complete recovery and the emergence of new ailments, altogether known as “long COVID,” is being reported in many survivors (17–19) (**Figure 1**). The clinical data presentations in COVID-19 patients have revealed some interesting facts; of the total cases, about 80% either are asymptomatic or have mild symptoms, while ~14% develop severe symptoms, such as pneumonia, ~5% develop critical symptoms, such as septic shock, respiratory failure, or multiorgan failure, and ~2% of the patients die of the disease (20). Fatality is comparatively much higher in the aged and persons with comorbidities (21, 22).

**Table 1:** Systemic diversity of clinical manifestations in coronavirus disease 2019 (COVID-19).

System	Symptoms	Study
**General**	Fever	(6, 7)
	Headache	
	Fatigue	
**Respiratory**	Dry cough	(6, 7)
	Difficulty to breathe	
	Congestion of nose	
	Runny nose	
	Sore throat	
**CNS and sensory organs**	Acute psychosis	(8–11)
	Loss of sense of smell	
	Loss of sense of taste	
	Loss of speech	
	Dizziness	
	Impaired consciousness	
	Stroke	
	Ataxia	
	Seizure	
	Impaired vision	
	Pink eye	
	Hearing loss, otalgia, vertigo, tinnitus	
**Cardiac**	Acute chest pain/pressure	(12)
	Arrhythmia	
	Heart failure	
**Digestive**	Nausea and vomiting	(6, 7, 13)
	Anorexia	
	Diarrhea	
	Abdominal pain	
**Renal**	Cloudy urine with frequent urge	(14)
**Musculoskeletal**	Myalgia	(6)
**Skin, hair, and nail**	Rash or discoloration of fingers or toes	(6, 15, 16)
	Hair fall and baldness	
	Red half-moon nail sign	

Based on the review of current literature, “long COVID” is characterized by symptoms of fatigue, headache, dyspnea, and anosmia. It is more likely to occur in aged, people with high body mass index, and the female sex, and the symptoms may persist for 4–12 weeks. Also, the COVID patients who experienced more than five symptoms during the first week of illness may have significantly higher chances of developing “long COVID” [odds ratio = 3.53 (2.76–4.50)] (17). A recent study has indicated that certain “long COVID” symptoms may persist beyond a year, more particularly, fatigue, dyspnea, and neurological symptoms, such as anxiety and depression (23).

## Virus–Host Interactions

### SARS-CoV-2 Entry Into Host Cells

**Figure 2:**
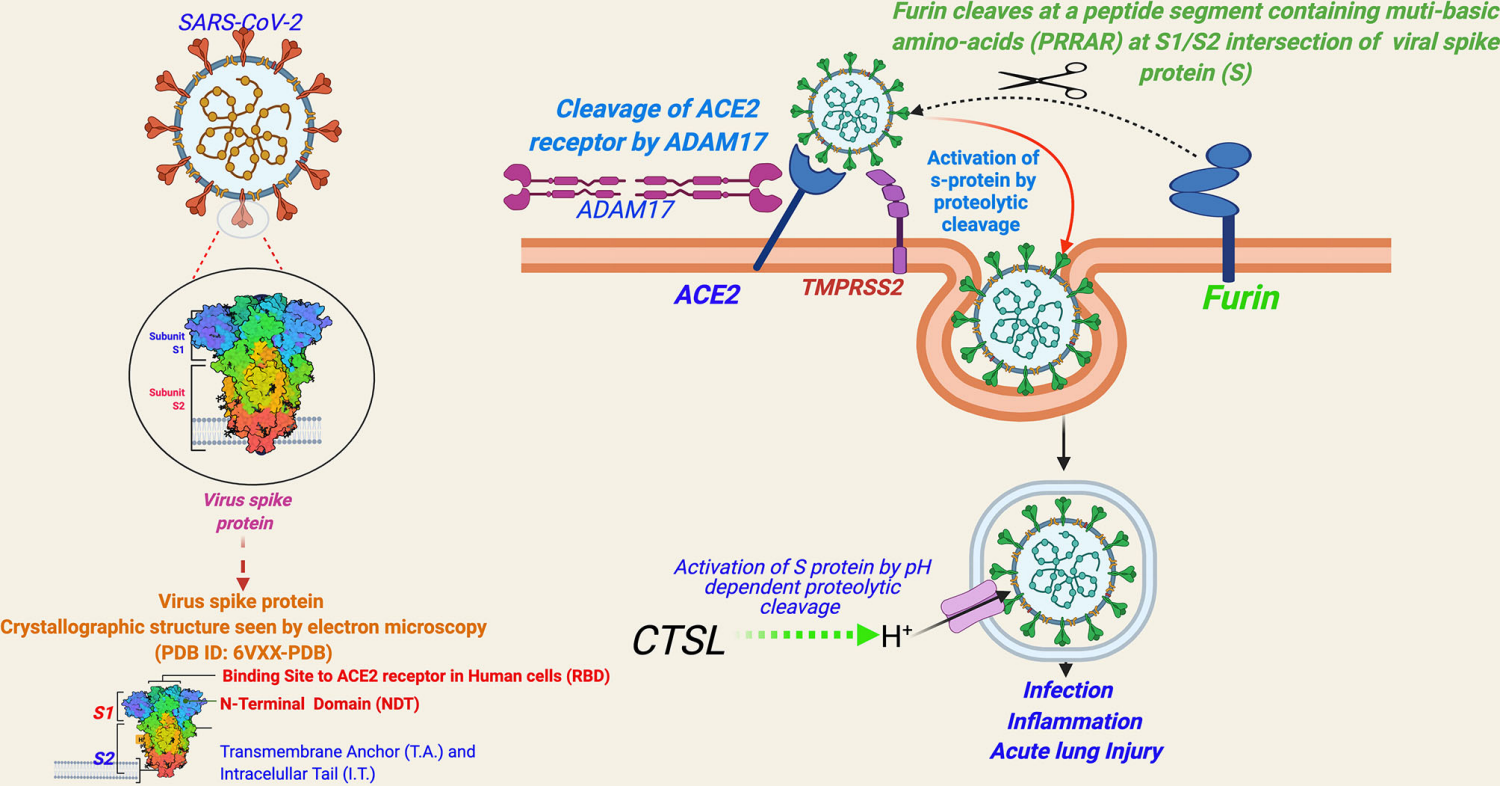
A schema for severe acute respiratory syndrome coronavirus 2 (SARS-CoV-2) entry into human cells. [Entry of SARS-CoV-2 into human cells is mediated by a cell surface receptor angiotensin-converting enzyme-2 (ACE2). ACE2 binds to receptor-binding domain (RBD) of SARS-CoV-2 spike (S) protein. Furthermore, to enter into the host cell, the priming of the viral spike protein (S) for its fusion to host cell membrane is done by host cell proteases, which involves cleavage of ‘S’ protein by the serine proteases, transmembrane serine protease 2 (TMPRSS2) or Cathepsin B or L (CTS-B or -L), and furin present in the host cell membrane. CTS-B or -L) acts primarily inside the endosomes. Furin cleavage site (PRRAR), present at the intersection of S1 and S2, is considered an evolutionary gain in SARS-CoV-2.

Entry of SARS-CoV-2 into human cells is mediated by a cell surface receptor angiotensin-converting enzyme-2 (ACE2) (24) (**Figure 2**). ACE2 binds to the receptor-binding domain (RBD) of SARS-CoV-2 spike (S) protein. Furthermore, to enter into the host cell, the priming of the viral spike protein (S) is considered essential for its fusion to host cell membrane, which involves cleavage of the “S” protein by serine proteases called transmembrane serine protease 2 (TMPRSS2) or by cathepsin B or L (CTS-B or -L) and furin present in the host cell membrane (1) (**Figure 2**) [Reviewed in (1)].

ACE2 has been a known host cell entry receptor for some other CoVs as well, such as SARS-CoV-1 and HCoV-NL-63. TMPRSS2 and CTS-L are also known for SARS-CoV-1 and other SARS-related viruses (1). However, the inclusion of furin seems to be an evolutionary gain in SARS-CoV-2 in comparison to other SARS viruses [Reviewed in (1)]. Furin cleavage needs insertion of a peptide segment, furin cleavage site (FCS), containing multi-basic amino acids (PRRAR) at the S1/S2 intersection of viral spike protein (S), which are not present in SARS-CoV-1 or other SARS-related viruses (25). Notably, FCS is known to be present in many other CoVs including MERS-CoV, HKU1-CoV, and OC43-CoV (26). The inclusion of FCS in the SARS-CoV-2 genome is speculated to be contributing to its high infectivity and transmissibility in comparison to other SARS viruses (25). FCS has also been observed in influenza viruses and is considered contributing to virulence (27). Currently, there is limited evidence whether FCS inclusion is contributing to the virulence of SARS-CoV-2 [Reviewed in (1)]. In a recent study, SARS-CoV-2 mutant lacking FCS in the spike protein was found to have reduced replication in Calu3 cells (a human respiratory cell line) and attenuated disease progression in a hamster pathogenesis model of COVID-19 (28).

Other than ACE2, concrete evidence for an alternative host cell entry receptor for SARS-CoV-2—neuropilin-1 (NRP1)—has been identified by recent studies (25, 26). NRP1 is abundantly expressed in multiple tissue types across the body, with very high expression in endothelial and epithelial cells, particularly, in the respiratory and olfactory epithelium. Cantuti-Castelvetri et al. (29) showed that NRP1 potentiates SARS-CoV-2 infectivity. Furthermore, Daly et al. (30) showed that the furin-cleaved S1 fragment of the spike protein can bind directly to cell surface NRP1, and blocking this interaction, using a small-molecule inhibitor or monoclonal antibodies, effectively reduces SARS-CoV-2 infection.

### Tissue Tropism and Organotropism

**Figure 3:**
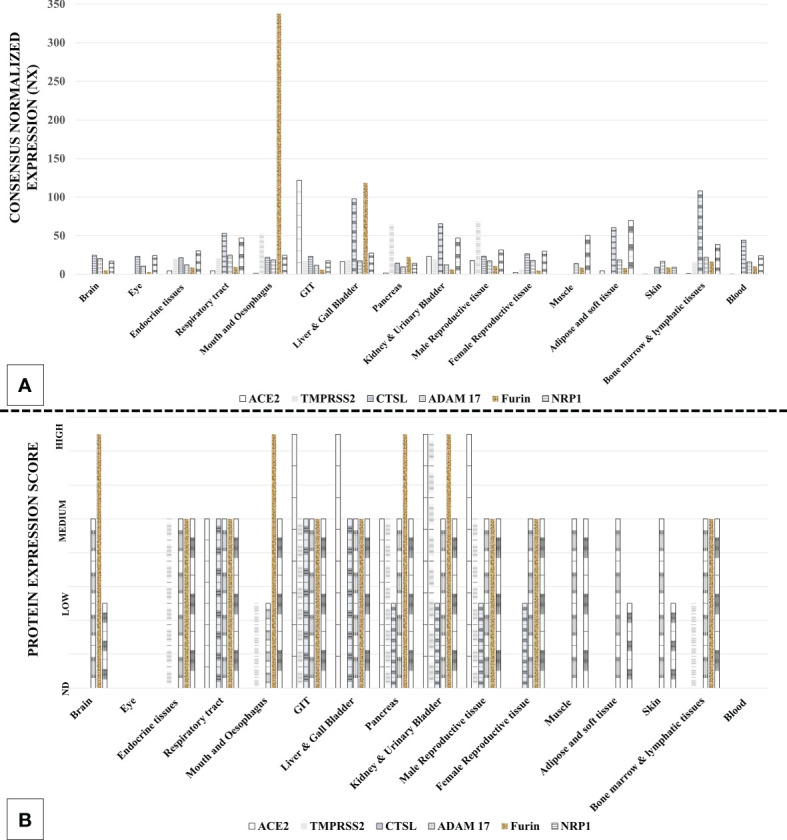
Expression of severe acute respiratory syndrome coronavirus 2 (SARS-CoV-2) host cell entry receptor and entry-associated proteases in human tissue types. **(A)** mRNA. **(B)** Protein. [Data source: Human Protein Atlas (https://www.proteinatlas.org/)].

SARS-CoV-2 host cell entry factors are widely expressed across the tissue types in humans (31–33). Additionally, ACE2 is co-expressed with TMPRSS2/CTS-L in many tissue types, which is an essentiality for SARS-CoV-2 infection (32, 33). The extensive tissue tropism of SARS-CoV-2 is reflected in the diversity of the symptoms in COVID-19 patients (5). Multiorgan tropism of the SARS-CoV-2 has also been confirmed in the studies involving histopathological observations in the postmortem tissue samples from the infected patients (34) and laboratory infection of human tissue organoids (35). **Figure 3** describes the tissue types that can get infected with SARS-CoV-2 based on the expression of host cell entry receptors and entry-associated host proteases.

### Hijacking of the Host Cell Machinery

Recent studies have unraveled SARS-CoV-2-driven molecular mechanisms hijacking host cell machinery, particularly, for the production of proteins and energy production mechanisms (36, 37). The *in vitro* studies have provided robust evidence for extensive phosphorylation of SARS-CoV-2 viral proteins by the host proteome involved in activation of the host cell kinases and growth factor receptor (GFR) signaling, thus facilitating hijacking of the host protein machinery. The very first such evidence presented by Bouhaddou et al. (38) showed that SARS-CoV-2 infection of the host cells promotes casein kinase II (CK2) and p38 mitogen-activated protein kinase (MAPK) activation and production of diverse cytokines, leading to the shutdown of cyclin-dependent kinase (CDK) 1/2/5 and cell cycle arrest (38). Authors also noted a unique feature of SARS-CoV-2 that was least known for the respiratory viruses: viral infection markedly induced unique CK2-containing filopodia protrusions, which contained budding viral particles. The filopodia protrusions appeared to facilitate the transfer of the infective virus across the host cells. This pattern of molecular pathway activation by SARS-CoV-2 could potentially explain the hallmarks of host tissue injury in severe COVID-19, such as acute inflammation and epithelial cell damage and vascular endothelial dysfunction. The other prominent evidence was secondly provided by Klann et al. (37), showing that SARS-CoV-2 infection of a human colonic epithelium cell line—Caco-2 cells—activated GFR signaling and its downstream pathways. Additionally, the authors showed that inhibition of GFR signaling prevented replication of SARS-CoV-2 in host cells (37). Furthermore, another recent study involving a mouse model of COVID-19 has suggested that SARS-CoV-2-induced systemic toxicity causes downregulation of expression of genes affecting the energy production mechanisms in the cells, such as oxidative phosphorylation and the tricarboxylic acid (TCA) cycle, and epigenetic (DNA methylation) changes in the vital organs (38).

SARS-CoV-2 non-structural proteins (NSPs), more particularly, NSP1, which binds to the 40S ribosomal subunit of the host cells, have been reported to cause the shutdown of mRNA translation in host cells (36). It has also been found to block retinoic acid-inducible gene I (RIG-I) and interferon-stimulated genes (ISGs), which are key mediators of host innate immune response in case of viral infections (39). Another SARS-CoV-2 protein, NSP16, in conjunction with NSP10, protects the virus from host innate immune response by methylating the 5′-end of the virus-encoded mRNAs (thus mimicking host cellular mRNAs) (40).

Apart from the above-stated mechanisms, a mimicry of the SARS-CoV-2 spike protein to a human epithelial cell ion-channel, thus hampering its physiological functions, has also been shown recently. Anand et al. (41) have shown that S1/S2 cleavage site of spike protein (S) of SARS-CoV-2 has a striking protein sequence similarity to the furin-cleavable peptide segment (FCS) on the human epithelial sodium channel α-subunit (ENaC-α). The mimicry of the viral spike protein to ENaC-α in host cells indicates that the virus can compete for the furin available in the infected host cells and thus may block proteolytic activation of ENaC-α. ENaC-α has established roles in the development of acute respiratory distress syndrome (ARDS) mediated through immune cell activation and cytokines/chemokines (42, 43), indicating that this mechanism may have a role in the pathogenesis of ARDS in severe COVID-19 patients.

## Dysregulation of Renin–Angiotensin–Aldosterone System (RAAS)—A Key Axis Maintaining Physiological Homeostasis

**Figure 4:**
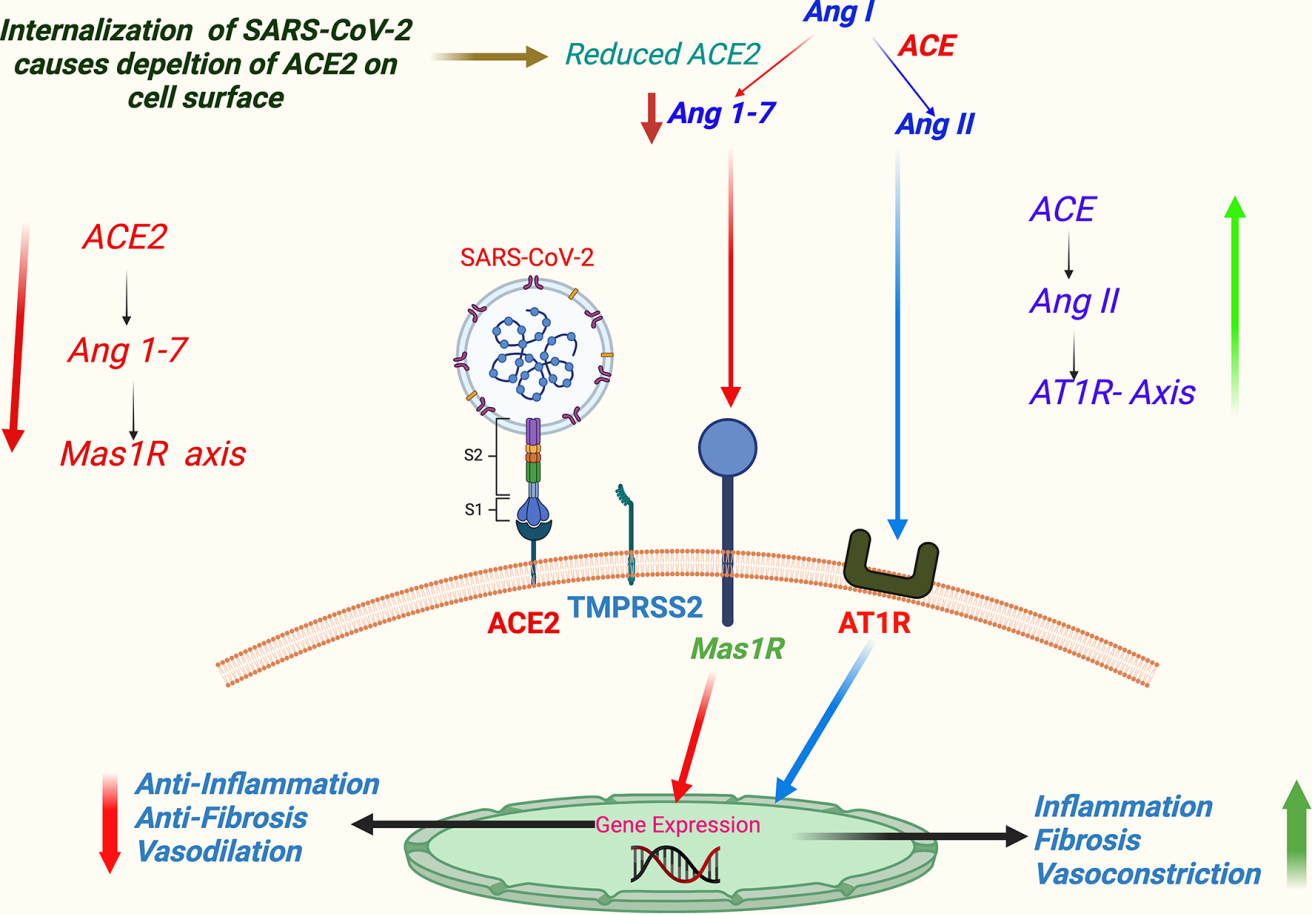
A schema of virus binding-induced ACE2-mediated dysregulation of RAAS in COVID-19. (SARS-CoV-2 host cell entry receptor ACE2 is an analog of ACE that performs a key step in regulation of RAAS—the conversion of Ang I to Ang II in lung epithelium. Ang II primarily acts through AT1 receptor. Alternatively, Ang II is metabolized to angiotensin 1-7 (Ang 1-7), which further acts through Mas 1R. Physiologically ACE/Ang II/AT1R axis keeps in balance with ACE2/Ang 1-7/Mas 1R axis. Supposedly, binding of SARS-CoV-2 downregulates ACE2 signaling, and consequently ACE/Ang II/AT1R axis gets an upper hand favoring vascular constrictions, tissue inflammation, and fibrosis.) Abbreviations: ACE, angiotensin converting enzyme; Ang I, angiotensin-1; Ang II, angiotensin-2; AT1R, angiotensin 1 receptor; COVID-19, coronavirus disease 2019; Mas 1R, Mas 1 receptor; RAAS, renin–angiotensin–aldosterone system; SARS-CoV-2, severe acute respiratory syndrome coronavirus 2.

Dysregulation of the renin–angiotensin–aldosterone system (RAAS) has been a characteristic feature in COVID-19 (44). RAAS regulates physiological hemodynamic balance involving all major organs, primarily liver, lung, heart, and kidney (22), and is also involved in the maintenance of electrolyte balance and vascular resistance; hence, it is a crucial determinant of systemic blood pressure and consequently cardiovascular health. SARS-CoV-2-induced dysregulation of RAAS is mediated through its host cell entry receptor ACE2, which is an analog of ACE that performs a key step in the regulation of RAAS—the conversion of angiotensin I to angiotensin II. An ACE/ACE2 balance is considered to be a crucial factor in maintaining an optimum functionality of the RAAS. The SARS-CoV-2 infection supposedly downregulates ACE2, but not ACE, and thus may be creating an imbalance of physiological ACE/ACE2 ratio (9, 17). Apart from that, a SARS-CoV-2 binding causes downregulation of ACE2 in RAAS components, which can induce activation and release of pro-inflammatory markers causing tissue injury (42). An ACE2-mediated dysregulation of RAAS is a key step in COVID-19 pathogenesis and contributes significantly to the comorbidity-associated mortality, vascular thrombosis, organ-specific morbidity, and multiorgan failures, which we discuss in detail afterward in this article in the related subsections. A schematic representation of virus binding-induced ACE2-mediated dysregulations of RAAS in COVID-19 is summarized in **Figure 4**.

## Multisystem Involvement in COVID-19: Understanding Underlying Mechanisms

The extensive distribution of the SARS-CoV-2 host cell entry factors in the human body indicates that the virus can potentially infect most of the organs and tissue types. Initially, it was perceived that the primary organ involved in COVID-19 pathogenesis is the lung; however, the accumulating body of evidence over time has established that COVID-19 is a systemic disease with extremely diverse manifestations (5) (**Table 1**). In many cases, the patients particularly present with the non-respiratory symptoms, involving single or multiple other organs, including the brain (5). A comprehensive review of the current literature suggests that the multisystem involvement in COVID-19 can be largely explained by the widespread distribution of SARS-CoV-2 host cell entry receptors, direct viral toxicity, dysregulated host immune response, involvement of RAAS and systemic hyper-inflammatory response against the infection, and macrovascular and microvascular thrombosis. The current empirical evidence on all these aspects, involved in the pathophysiology of COVID-19, about the key physiological systems is mentioned below.

### Respiratory System

SARS-CoV-2 cell entry receptors are expressed by multiple cell types along the respiratory tract. In the upper respiratory tract, ciliated nasal secretory epithelial cells co-express ACE2 and TMPRSS2 in abundance (32). In the lower respiratory tract, ACE2 and TMPRSS2 are co-expressed, more abundantly, in type 2 pneumocytes than type 1; additionally, both receptors are expressed in the goblet, club, enteroendocrine, bronchial cells and endothelial cells of the pulmonary vasculature (32, 33, 44). Though the mild respiratory symptoms can be attributed to infection in the upper respiratory tract, the characteristic SARS or ARDS is caused when lung cells, primarily type 2 pneumocytes and pulmonary vascular endothelial cells, are infected (45). Severe lung inflammation may ensue with infection of these cells first by the involvement of resident macrophages and then by the recruitment of peripheral macrophages and other immune cells, such as neutrophils and T cells (20, 45). The hampered production of surfactants (by type 2 pneumocytes) and consolidation of the accumulating exudates cause a collapse of the alveoli and induce pneumonic changes (46). Uncontrolled initial inflammation may cause the release of more pro-inflammatory cytokines and recruitment of peripheral immune cells, thus in a vicious cycle inducing more tissue injury (20). Pulmonary vascular endothelialitis and subsequent thrombosis, primarily of the microvessels, marked by elevated blood levels of fibrin degradation products (FDPs), D-dimer, and prothrombin time (PT) also contribute effectively to the lung pathology (20, 45, 47).

### Cardiovascular and Renal Systems

Cardiac myocytes, endothelium of coronary vessels, and fibrocytes are known to have significant expression of ACE2 and serine proteases, primarily TMPRSS2 (48). Cardiac symptoms may arise because of direct myocardial injury caused by the virus (49, 50). Additionally, SARS-CoV-2-driven RAAS dysregulation may cause increased incidences of thromboembolism, and the hypertensive episodes may be ensued by persistent constriction of systemic and coronary vessels (51, 52). Furthermore, an inflammation in the coronary arteries may speed up the formation of plaques, causing blockage and thus the ischemic changes leading to heart failure (12, 53). Electrolyte imbalance induced by the RAAS dysregulation may be another mechanism leading to heart ailments, especially hypokalemia can cause hyperpolarization of the cardiac myocytes leading to arrhythmia (53). The hypokalemia in COVID-19 may also be caused by direct viral-mediated directed myocardial injury leading to decreased cardiac output, thus activating aldosterone-mediated renal excretion of potassium ions (53, 54).

Renal involvement is highly indicative in COVID-19, taking into account the significant expression of SARS-CoV-2 host cell entry receptor ACE2 and associated proteases, particularly TMPRSS2 in epithelial cells of renal tubules and podocytes of the glomeruli (33, 55). In that line, acute renal involvement and the evidence of virus-induced injury, including the presence of viral particles in the renal tubules upon postmortem histopathological examination, have been reported (34). However, the prevalence of direct renal/kidney involvement in COVID-19 is low if compared to the other key systems/organs (56). Renal injury may also be secondary to ARDS and cytokine storm-induced sepsis. It may also be caused by ACE2-mediated dysregulation of RAAS or due to multiple iatrogenic causes, such as a fallout of intensive care unit management or mechanical ventilation and nephrotoxic effects of the drugs (57).

### Digestive System

Prominent digestive symptoms have been noted in many COVID-19 patients (6, 13), and SARS-CoV-2 particles have been detected in the cells, with evidence of inflammatory lesions, in the gastrointestinal tissues, in multiple postmortem studies (34, 58). SARS-CoV-2 host cell entry factors (ACE2 and TMPRSS2) are enriched in intestinal tissue (13), and successful viral invasion has been shown in human intestinal organoids (59, 60) and animal model studies, including in primates (61, 62).

Whereas, at present, there is sufficient evidence confirming that SARS-CoV-2 can infect the gastrointestinal tract (GIT), how the virus reaches this is yet not well understood. A fecal–oral route of the viral entry is the most plausible explanation for this (62). Shedding of infectious SARS-CoV-2 in feces was detected in some COVID-19 patients, although it has not been a regular finding (62). It is intriguing how SARS-CoV-2 survives extremes of the pH within the digestive system milieu (gastric, 1.5–3.5; pancreatic, 7.5; bile acid, 7–8) while passing along the length of the GIT. SARS-CoV-2 is known to survive at a wide range of pH values at room temperature (pH 3–10) (13). RNA viruses like influenza A and B (when swallowed) can survive the extremes of pH and maintain infectivity with the help of the mucus cover lining the GIT, allowing their safe passage and even excretion in feces (63). As mucus cells are abundant throughout the GIT, they can contribute to the carriage and survival of SARS-CoV-2 (13).

Of note, the healthy intestinal mucosa may not be well conducive for the entry of the virus owing to the presence of a unique multilayer barrier system, though the presence of a prior inflammatory condition that disrupts the mucosal barrier may make that permissive (13, 64). Additionally, inflammatory conditions in GIT may support entry of the virus by inducing the expression of ACE2 in the mucosal epithelium (13). Thus, a prior intestinal inflammatory condition, like inflammatory bowel disease (IBD), may increase the susceptibility to SARS-CoV-2 infection through fecal–oral transmission (13). The composition of the gut microbiome of the subject is another important factor influencing contractibility and severity of symptoms (65) (discussed further in subsection *Gut Microbiome* under section *Host Factors Affecting Transmissibility, Severity, and Patient Outcomes*).

Other than the fecal–oral route, an alternative route of viral entry to the GIT cells can be through the tissue microvasculature (13). As per current evidence, the blood is known to carry and transmit SARS-CoV-2, and vascular endothelium expresses ACE2 and TMPRSS2 abundantly (45, 66). Viral infection-induced inflammation and tissue injury including rupture of the intercellular junctional complexes may make the small vessels permeable for passage of the virus (67).

Other than GIT, tissue injury has also been noted in the other components of the digestive system, *viz.*, liver and biliary duct, and pancreas (34, 68). Elevation of the liver enzymes has been a frequent finding in severe cases of COVID-19 (68). Paradoxically, there has been no clear evidence of a direct viral invasion to the liver tissue (34); however, model studies in human tissue organoids have shown that SARS-CoV-2 can infect hepatocytes, as well as cholangiocytes in the biliary ductal epithelium (35). Of note, proteomic and transcriptomic studies suggest that the expression of key viral host cell entry receptor ACE2 is primarily limited to cholangiocytes (69). Observing the limited expression of ACE2 in hepatic tissue, there remains a plausibility that any hepatic impairment in COVID-19 may be primarily not due to direct viral injury but indirect reasons, such as systemic hyper-inflammation, dysregulated immune response, and thrombosis of microvessels. Pancreatic involvement in COVID-19 is intriguing, as the severe glycemic impairment including the onset of new diabetes (also discussed later in subsection *Comorbidities*) has been noted in the patients (70, 71); however, a clear evidence, showing that COVID-19 can do this on its own accord, is still lacking [Reviewed in (72)]. Although there has been vague evidence in high-throughput proteomic and transcriptomic studies on the secretion of ACE2 by the pancreatic components (13), the studies involving single-cell RNA sequencing clearly showed significant expression of ACE2 in the beta cells secreting insulin, and SARS-CoV-2 was shown to be capable of infecting endocrine cells (alpha and beta) of the human pancreatic organoids (35).

### Nervous System

Mild neurological symptoms in most of the cases, like headache, nausea, vomiting, dizziness, loss of the sense (smell and taste), and, in certain cases, severe symptoms like ataxia, convulsions, altered consciousness, ischemic or hemorrhagic stroke, acute disseminated encephalomyelitis (ADEM), meningitis, encephalitis, and rarely Guillain–Barré syndrome variants (Miller Fisher syndrome and polyneuritis cranialis) (8, 73–76), and new onset of psychotic symptoms (77) have been reported in COVID-19. Autopsy studies in COVID-19 deceased have shown widespread brain lesions (mostly reflecting acute hypoxic-ischemic injury) (78). Although rare, the viral RNA is also detected in brain tissue (78) and the cerebrospinal fluid (CSF) (74, 79) of the COVID-19 deceased.

How SARS-CoV-2 enters the central nervous system and mediates the pathogenesis of neurological symptoms in COVID-19 patients is now getting explained in light of emerging facts (80). The most likely route of viral spread to the brain is transneuronal spread through the olfactory nerves. In association, the hematogenous route after breaching the blood–brain barrier (BBB) is also possible (67). The studies noted significant expression of ACE2 and TMPRSS2 in cells of olfactory epithelium and myelin (oligodendrocytes) and neurovascular endothelium in humans (80). In addition, model studies in transgenic mice, which expressed human ACE2, have demonstrated the intracranial spread of SARS-CoV-2 to the parts of the brain *via* the olfactory pathway following intranasal inoculation (80). The mounting pieces of evidence strongly favor the neuroinvasive potential of SARS-CoV-2. Furthermore, the new onset of psychotic symptoms in some patients of COVID-19 (77) indicates a synaptic pathology occurring in the brain regions associated with executive functions.

Studies suggest that neurological symptoms (including the loss of smell and taste) may arise due to the direct neuropathic effect of the virus. Alternatively, it can also be an indirect effect of cytokine-induced neuroinflammation or immune cell-mediated (81, 82) effect on neurons (or glial cells) or endothelial cells of cerebral microvasculature inducing cellular apoptosis and increased vascular permeability and edema of the related brain tissue.

The mediation of SARS-CoV-2 infection in the brain through the recently proposed alternative receptor—NRP1—is a strong plausibility. Interestingly, NRP1 has significant expression in the human brain including olfactory neurons. The proof of the concept for NRP1-mediated SARS-CoV-2 entry into the brain is received from a recent study by Cantuti-Castelvetri et al. (29) demonstrating the presence of SARS-CoV-2 spike protein in NRP1-expressing neurons and endothelial cells of capillaries and medium-size vessels of olfactory bulb and tract in brain autopsy specimens from COVID-19 deceased. The authors also demonstrate in mice, following intranasal administration, NRP1-dependent delivery of virus-size nanoparticles (80 nm diameter) to the olfactory epithelium and neuronal cells of the olfactory bulb and central nervous system (cortex) that signifies the olfactory pathway as a route for SARS-CoV-2 entry into the brain (29).

The neurological symptoms may also arise due to several other reasons, such as metabolic encephalopathy arising from dysfunction of the vital organs (like lung, liver, and kidney) (7) or increased risk of neurovascular thrombosis in patients with severe COVID-19 (83), associated comorbidities, or age-related neurovascular pathologies.

### Other Systems and Tissue Types

COVID-19 is said to involve not only the key physiological systems (discussed above) but also almost all other systems (5), including sensory organs, *viz.*, eyes (11) and ears (10), and integument, *viz.*, skin, hair, and nails (15, 16). The pathological involvement of most of these systems/tissue types can also be predicted based on the expression of viral host cell entry factors (31) (**Figure 2**). Multiple mechanisms can be responsible for the injury of the SARS-CoV-2-infected tissue, *viz.*, a direct injury caused by the viral cytotoxicity (1), endothelial dysfunction mediated through the viral host cell entry receptor ACE2 and consequent vascular thrombosis (45), or inflammatory damage owing to an excessive immune response against the infection (20), or it can be due to a virus-independent mechanism, such as certain tissue-specific immunopathologies that are currently not well explained (84).

## Host Immune Response to SARS-CoV-2 Infection

SARS-CoV-2 infection ensues varying immune responses in the different individuals, which decide the severity of the symptoms (85). Some individuals get away with the mildest of the symptoms or remain completely asymptomatic; in contrast, in others, the viral infection leads to severe disease manifestations resulting in ARDS and multiorgan failure (20). Similar to other viral infections, a cytokine-mediated innate defense is the first immune response in the infected individuals. A peculiar form of systemic hyper-inflammatory state, characterized by very high levels of pro-inflammatory cytokines, known as “cytokine storm” (CS) is commonly observed in patients with severe COVID-19 (20). Of note, CS is not peculiar to COVID-19, and it has been a characteristic finding in the severe stages of many other respiratory viral diseases, such as SARS, MERS, and influenza (86). However, SARS-CoV-2-induced CS is different compared to other respiratory viruses, as SARS-CoV-2 does not necessarily induce a common cytokine signature, such as interleukin (IL)-2, IL-10, IL-4, or IL-5 (87). In COVID-19, CS is characterized by a particular set of cytokines highly increased in the serum of the patients, such as IL-2, IL-7, granulocyte-macrophage colony-stimulating factor (GM-CSF), granulocyte colony-stimulating factor (G-CSF), Interferon gamma-induced protein 10 (IP10), macrophage inflammatory protein 1-α (MIP1-α), monocyte chemoattractant protein 1 (MCP-1), Tumour necrosis factor α (TNFα) and Interferon γ (IFN-γ). The circulating concentrations of chemokine (C-X-C motif) ligand-10 (CXCL10), chemokine (C-C motif) ligand 2 (CCL2), IL-2R, IL-6, TNFα, C-reactive protein (CRP), and ferritin are significantly higher in those needing admission to intensive care units (ICUs) (87). Furthermore, it is targeted particularly to the dysregulation of the type-I IFN response and its downstream cytokines (87).

**Figure 5:**
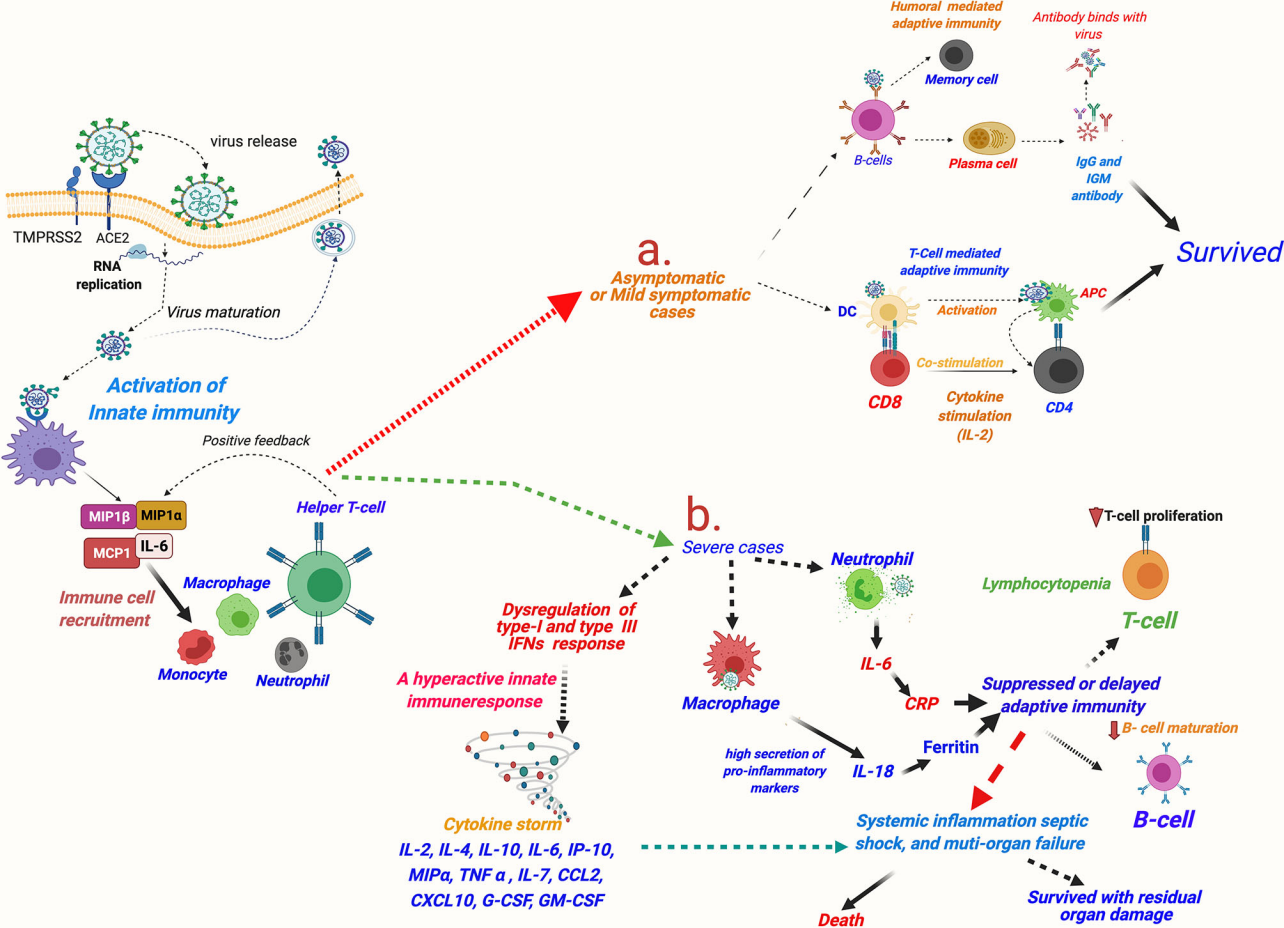
A schematic description of immune responses in asymptomatic and mildly symptomatic and severe cases of COVID-19. SARS-CoV-2 invasion mediated by host cell entry receptor and entry-associated proteases leads to activation of innate immune response and recruitment of circulating immune cells in lung epithelium. Furthermore, immunological response is varied in asymptomatic/mildly symptomatic and severe cases of COVID-19 patients: **(A)** asymptomatic and mildly symptomatic cases, an optimum activation of T-cell and humoral-mediated adaptive immune response leads of cure of the patients; **(B)** in severe cases, a hyperactive innate immune response leading to cytokine storm and consequently killing of T cells and delayed/or suppressed B cell-mediated humoral response resulting in very poor patient outcomes is observed. MIP, macrophage inflammatory protein; MCP, monocyte chemoattractant protein; IL, interleukin; G-CSF, granulocyte colony-stimulating factor; GM-CSF, granulocyte-macrophage colony-stimulating factor; IFN, interferon; TNF-α, tissue necrosis factor-α; CCL2, chemokine (C-C motif) ligand 2; CXCL10, chemokine (C-X-C motif) ligand-10; COVID-19, coronavirus disease 2019; SARS-CoV-2, Severe acute respiratory syndrome coronavirus 2.

Researchers worldwide are endeavoring to understand the exact reasons for the heightened innate immune response seen in COVID-19 patients. A review of the existing literature suggests that it may be partly explained by the known facts about unique host–pathogen interactions occurring in respiratory viruses [(Reviewed in (20)]. The first defense of the host against a viral infection is marked by the rise of the key innate immune response molecule—IFNα (20). Similar to SARS and MERS (88), a delayed IFNα response has been observed in COVID-19, which indicates it being a virus-mediated mechanism facilitating the viral cell entry. A delayed immune response would facilitate entry of the virus in the lung epithelium and that in turn would lead to an intense inflammatory response caused by incremental recruiting of the wide repertoire of innate immune cells (89). Interestingly, different from the other respiratory viruses, such as SARS-CoV, MERS-CoV, and influenza virus type A (IVA), a very distinctive strategy seems to be used by SARS-CoV-2 (90). SARS-CoV-1, MERS-CoV, and IVA cause complete dampening of the IFN or chemokine (the pro-inflammatory cytokines with chemoattractant properties)-mediated innate immune response at the time of infection. In contrast, SARS-CoV-2 showed a dampened IFN (type I, and also type III) response but paradoxically highly elevated chemokines (89) (**Figure 5**). The dampening of early IFN response against SARS-CoV-2 infection is mediated by a viral protein ORF3b (90). This unique dichotomy in the host innate immune response in COVID-19 can promote viral invasion of the respiratory epithelium (and epithelial cells at other sites) and facilitate their viral replication. High viral load in the infected host epithelial cells in turn can induce tissue injury and, as a consequence, incremental secretion of chemokines and recruitment of circulating immune cells (**Figure 5**). A hyperactive innate immune response would paradoxically hamper viral clearance and promote further viral replication (1).

Optimum T cell-mediated and humoral-mediated adaptive immune responses are usual in asymptomatic and mild symptomatic cases. Conversely, a subthreshold and delayed protective T cell-mediated and humoral-mediated adaptive immune responses in symptomatic patients are pronounced in the patients with severe COVID-19 in the initial period. However, the survivors showed a robust and durable adaptive immune response (91) [Reviewed in (92)].

Interestingly, Th1 cells, a type of activated CD4 T cells, and lung tissue-resident memory-like Th17 (Trm 17) cells characterized by potentially pathogenic cytokine expressions of IL-17A and GM-CSF are observed to be higher in patients with severe disease in comparison to those with moderate disease (93). Interestingly, GM-CSF was found distinctively raised only in severe cases of COVID-19 and not in influenza when both conditions were compared (94).

Lymphocytopenia, particularly for T cells and more intensively for CD8 T cells, is a common observation regardless of the disease stage and severity in COVID-19 cases (22). However, lymphocytopenia correlated with the severity of the symptoms, and very low numbers of T cells predicts poor patient outcomes (95, 96). In contrast, in most of the patients with mild or moderate symptoms and with stable lymphocyte count, neutralizing antibodies [immunoglobulin G (IgG)] for a viral spike or other proteins appear nearly between 18 and 21 days and outcomes are fairly good. Molecular reasons for the killing of T cells in COVID-19 are little understood (97). Of note, T cells show less expression of SARS-CoV-2 entry receptors, providing a hint that the virus-mediated T-cell demise in the infected individuals may be occurring by some other mechanism rather than through viral receptor signaling (85). SARS-CoV-2-mediated atrophy and lesion of the human lymphoid tissues, such as spleen and lymph nodes, has also been evidenced in recent studies (58, 98, 99). The analysis of laboratory parameters of COVID-19 patients suggests that high levels of pro-inflammatory markers, or the cytokine storm, may be a potential reason for the killing of the T cells (100). TNF-α, IL-6, IL-8, and IL-10 levels are found to be significantly increased and negatively correlate to T-cell counts in severe COVID-19 (100). That high serum levels of TNF-α and other cytokines induce apoptosis of T cells is well known in inflammatory diseases, including SARS and MERS (101).

Based on the review of the current evidence (discussed above), the improved replication in host cells resulting in greater viral load and virus-mediated dysregulation of innate as well as adaptive host immune response seems to be the most important mechanism contributing to higher virulence and mortality risk to SARS-CoV-2 with respect to influenza and other BCoVs (SARS-CoV-1 and MERS-CoV). Hence, along with reducing viral replication with antiviral drugs, managing dysregulated host immune response, specifically innate, using immunomodulators, anti-inflammatory cytokines, and pro-inflammatory cytokine/or pathway-targeted antibodies becomes an important therapeutic strategy in COVID-19 [(Reviewed in (20)]. Noteworthy, apart from being a result of the SARS-CoV-2-induced dysregulation, the hyperactive innate immune response may be contributed by the factors intrinsic to the subject (102, 103). Specifically, in some young and healthy adults with no obvious comorbidity, the development of severe COVID-19 and subsequent mortality strongly indicates the presence of a subject-specific vulnerability (103). This is likely that such individuals may have a genetic or immunophenotypic predisposition for developing severe illness (102, 103). We discuss this issue in greater detail in the section *Host Factors Affecting Transmissibility, Severity, and Patient Outcomes* (subsection *Genetic and Immunophenotypic Factors*).

## Pathophysiology of Vascular Thrombosis and Multiorgan Failure

Thrombosis of the macrovessels and microvessels across the organs, particularly in the pulmonary vasculature, has been a prominent manifestation in severe COVID-19. Vascular thrombosis has been linked to the genesis of multiorgan failures and associated with very poor disease outcomes, including enhanced mortality. Multiple reasons have been suggested for the etiology of vascular thrombosis in COVID-19, such as toxicity of viral proteins, high levels of pro-inflammatory markers and cytokine storm, the prothrombotic impact of severe illness, and the iatrogenic causes (47, 104, 105). However, a SARS-CoV-2 binding-induced ACE2-mediated mechanism seems to be at the root of all these mechanisms (45, 105, 106). SARS-CoV-2 host cell entry factors ACE2 and TMPRSS2 co-express in endothelial cells of human blood vessels and microvasculature (45) and blood cell components particularly platelets (106). SARS-CoV-2 binding-induced downregulation of ACE2 can induce activation and release of pro-inflammatory markers and thus can cause injury of vascular endothelium (45, 107, 108). Additionally, a reversal of ACE/ACE2 ratio in the vascular endothelium dysfunction can also induce thrombosis (45). A lower ACE/ACE2 ratio in the vascular endothelium is known to prevent prothrombotic cascade from activation by catalyzing the degradation of angiotensin I (Ang I) to inactive angiotensin 1-9 and angiotensin II (Ang II) to angiotensin 1-7 with antiproliferative, antifibrotic, and vasodilatory functions through G protein-coupled Mas receptors (45, 105). Conversely, a higher ACE/ACE2 ratio allows increased conversion of Ang I to Ang II and binding of the latter to its type 1 (AT1) receptors, thus can induce vasoconstriction, inflammation, and fibrosis, and eventually vascular thrombosis (45, 105). Furthermore, SARS-CoV-2-mediated downregulation of ACE2 in vascular endothelium can activate the kallikrein–bradykinin pathway, inducing platelet aggregation and leaking of the vessels that can further add to the thrombotic episodes (45). Alternatively, direct binding of SARS-CoV-2 to ACE2 expressed on the platelets may also induce platelet aggregation and consequent thrombosis (106).

Plausibly, the activation of the host “complement system” caused by viral protein toxicity and cytokine-induced systemic inflammatory response may significantly add to the pathogenesis of vascular thrombosis (20). Gao et al. (109) recently demonstrated in mice that the N proteins of the BCoVs (SARS-CoV, MERS-CoV, and SARS-CoV-2) bound to Mannan-binding lectin serine protease-2 (MASP-2) (a key serine protease involved in complement activation) that led to aberrant activation of the host complement system and consequently microvascular thrombosis and aggravated inflammatory lung injury. Interestingly, the viral activation of the complement system was reversed with the application of MASP-2 antibodies (109).

In the vessels of the lung, primarily of the microvessels, neutrophilia and the formation of extracellular neutrophils traps (NETs) are being frequently observed in severe cases. The NETosis activation leads to increased concentrations of intracellular reactive oxygen species (ROS) in neutrophils, which induces endothelial dysfunction and activates coagulation pathways (both extrinsic and intrinsic), thus it can add to the ongoing event of vascular thrombosis. Hypoxia-induced hyperviscosity and upregulation of the HIF-1α (hypoxia-inducible factor 1 alpha) signaling pathway can be additional factors contributing to the thrombosis (5).

Vascular thrombosis added with the direct viral protein toxicity of the infected tissue and RAAS dysregulation-mediated hemodynamic imbalance, systemic hyper-inflammation, and toxic shock syndrome arising from the cytokine storm may culminate in multiorgan failure (105, 110, 111). The presence of the comorbidities and host-specific vulnerability for the severe disease symptoms may further contribute to the pathogenesis of the multiorgan failures (21, 112).

**Figure 6:**
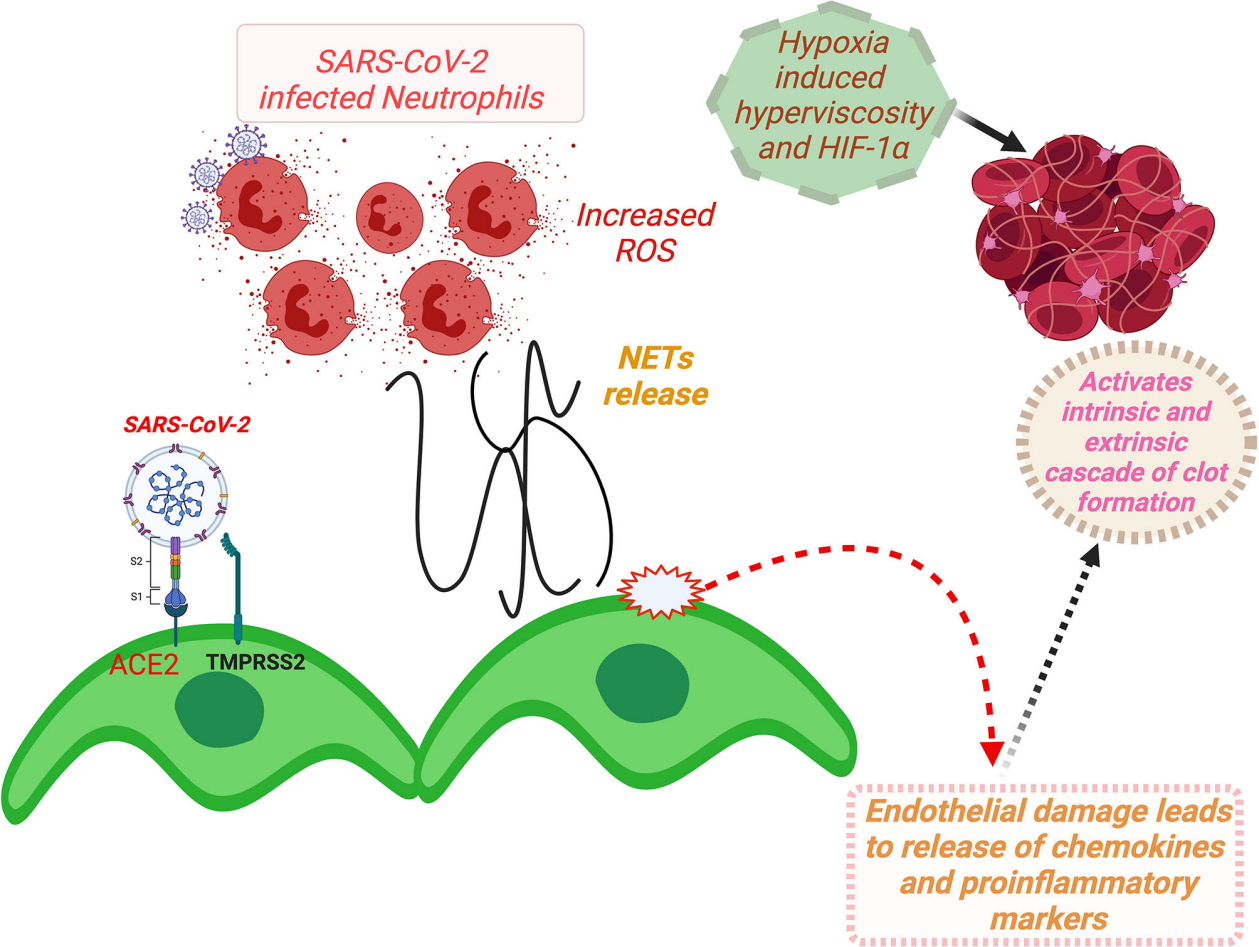
A schema for ACE2-mediated dysfunction of vascular endothelium leading to thrombosis in COVID-19 patients. (Binding of SARS-CoV-2 to ACE2 receptor expressed at the vascular endothelial cell surface leads to internalization and replication of the virus inside the cell and consequently endothelial dysfunction that activates prothrombotic cascade. Additionally, SARS-CoV-2 binding induces downregulation of ACE2, resulting in imbalances of ACE/ACE2 ratio, and dysregulation of RAAS, favoring prothrombosis. Both of these stated mechanisms in consequence also induce activation and aggregation of the platelets, altogether culminating in intravascular thrombosis. Furthermore, NETs that cause increased concentrations of intracellular ROS in neutrophils inducing vascular endothelial dysfunction and activation of coagulation pathways. Furthermore, hypoxia-induced hyperviscosity and upregulation of the HIF-1α signaling pathway can be contributing to the vascular thrombosis.) HIF-1α, hypoxia-inducible factor 1alpha; NETs, neutrophil extracellular traps; ROS, reactive oxygen species; ACE2, angiotensin-converting enzyme-2; TMPRSS2, transmembrane serine protease 2; COVID-19, coronavirus disease 2019; SARS-CoV-2, severe acute respiratory syndrome coronavirus 2; RAAS, renin–angiotensin–aldosterone system.

A schematic description for ACE2-mediated dysfunction of vascular endothelium involving RAAS and consequent thrombotic incidences and development of multiorgan failures in COVID-19 patients is summarized in **Figure 6**.

## Host Factors Affecting Transmissibility, Severity, and Patient Outcomes

Specific population groups, primarily men, aged, and those suffering from comorbidities, have been affected more aggressively by COVID-19 (21). The reasons why the disease affects more these specific population groups are now being gradually unraveled. Emerging scientific evidence indicates the multiple factors intrinsic to the host that are responsible for such poor outcomes in selected individuals (21). In this section, we are discussing, in brief, the current empirical evidence on the pathological basis of involvement of the key host intrinsic factors, which have been found to significantly influence transmissibility, severity, and patient outcomes for COVID-19.

### Age

Higher disease severity and mortality in individuals aged >50 years, more particularly in the elderly, are common observations in COVID-19 (113, 114). Several biological mechanisms have been suggested for this (115). The immunosenescence in elderly individuals could be the primary reason (116). The availability of naive T cells, the ratio of CD4/CD8 T cells, and T regulatory (Treg) cells decrease with aging (117). Plausibly, the immunosenescence compromises the response against a new pathogen, such as SARS-CoV-2 (118). Furthermore, the protective inflammatory response becomes worse with aging (116). In addition, elderly persons are most likely to have serious comorbidities (115).

In contrast, in the pediatric age group, a lesser number of cases and reduced fatality have been reported. Surprisingly, a rare multisystem inflammatory syndrome in children (MIS-C) has been reported across the world in this age group (119). MIS-C presents 4–6 weeks after SARS-CoV-2 infection as high fever and organ dysfunction, and patients show strongly elevated markers of inflammation. The pathogenesis of MIS-C is not yet clearly known. It has clinical features overlapping with Kawasaki disease suggestive of its being a vasculitis of an autoimmune etiology (120).

### Sex

Significantly higher disease severity and mortality have been observed in men as compared to women (121). A breakdown of the current global sex-disaggregated data shows that for every 10 women, respectively, 10 men are infected, 12 men are hospitalized, 17 men are admitted to ICU, and 13 men are dead (https://globalhealth5050.org, dated 08/10/2021) (121).

The causal basis of why men are affected more in COVID-19 is yet not well understood. Various reasons have been suggested for this [Reviewed in (122)]. Higher expressions of SARS-CoV-2 host cell entry factors, ACE2 and TMPRSS2, in reproductive organs of men (123–125) and androgenic regulation of TMPRSS2 (126) are suggested biological reasons for poor outcomes in men. In contrast, X-linkage and the estrogen-mediated regulation of multiple immune response genes including IFN type 1 and viral sensor TLR-7 could be the salient reasons why outcomes are comparatively better in women (127, 128).

### Comorbidities

Comorbidities have been the most significant host factors contributing to COVID-19 severity. The most frequent contributors to COVID-19 have been respectively cardiovascular diseases, diabetes, chronic respiratory disease, hypertension, and obesity [Reviewed in (129)].

Hypertension and obesity may independently predict patient outcomes in COVID-19 (130, 131), and preexisting diabetes may increase the risks of having severe/critical COVID-19 illness and in-hospital mortality by, approximately, 2-fold and 3-fold, respectively (129, 132). The reasons for increased vulnerability for COVID-19 in the presence of comorbidities are yet not much understood. Plausibly, the residual damage and dysregulation of the pulmonary physiology, and also of the other vital organs, in the comorbid patients add to the COVID-19-induced pathology (129). A SARS-CoV-2 host cell entry receptor ACE2-mediated dysregulation of RAAS can also be implicated in this (45).

Obesity sets a state of chronic systemic inflammation and also changes the phenotypes of immune cells from anti-inflammatory to pro-inflammatory (Th2 to Th1 CD4 T cells and M2 to M1 macrophages) and induces increased secretion of pro-inflammatory adipocytokines, such as leptin, and decreased secretion of anti-inflammatory adipokines such as adiponectin, favoring a severe inflammatory response against any new infection (133). Thus, obesity may aggravate the COVID-19 pathology by inducing an early overwhelming inflammatory response against the viral infection, such as cytokine storms (134).

Prominent glycemic changes including the new onset of diabetes and increased complications in some COVID-19 patients have been noted in recent studies (72, 135). Increased mortality linked with diabetic complications has been frequent in patients with COVID-19 (132). SARS-CoV-2 can invade insulin-producing pancreatic islet cells (35). In addition, ACE2 mediated downregulation of sodium-glucose co-transporter 1 (SGLT1) in intestinal epithelium prevents hyperglycemia in rat models of diabetes mellitus (13). SARS-CoV-2-mediated downregulation of ACE2 expression can eventuality lead to upregulation of SGLT1, thereby precipitating hyperglycemia (13). Apart from the intestine, SGLT1 is also expressed in other human tissues like the proximal tubule of the kidney, heart, and liver (proteinatlas.org/ENSG00000100170-SLC5A1/tissue). Thus, an ACE2-mediated invasion of pancreatic islet cells and/or dysregulation of SGLT1 in intestinal epithelium may be plausible mechanisms for the new onset of diabetes in COVID-19 patients.

### Genetic and Immunophenotypic Factors

Polymorphic variants are known for key SARS-CoV-2 host cell entry receptor ACE2 and associated host proteases, such as TMPRSS2 (136, 137) and furin (138). Possibly, some of these polymorphic variants of SARS-CoV-2 receptors may be more common in people showing low or high vulnerability for getting infected, or severity of symptoms and mortality, with COVID-19 (137).

Studies suggest that mutations in host viral sensors and immune genes may be a reason for increased vulnerability for developing severe COVID-19. A mutation of *toll-like receptor-7 (TLR-7)*—a viral sensor (including for the coronaviruses) on host cells—is found to be associated with severe COVID-19 (102). Another study has reported that at least 3.5% of patients with life-threatening COVID-19 pneumonia had known mutations for immune response genes, *viz.*, *interferon regulatory factor 7 (IRF7)* and *interferon (IFN)-alpha receptor 1 and 2 (IFNAR1 and 2)*, *TLR3*, TIR domain-containing adapter molecule 1 (TICAM1), *TANK-binding kinase 1(TBK1)*, and *IFN regulatory factor 3 (IRF3)* (139).

Two genomic regions have been found particularly associated with severe COVID-19: one region on chromosome 3 (locus 3p31.21) containing six genes and another region on chromosome 9 (locus 9q34.2) representing ABO blood groups (21, 140). Interestingly, the genetic variants on chromosome 3 [45,859,651–45,909,024 (hg19)] have entered the human population by a gene flow from archaic non-*Homo sapiens* hominids—Neanderthals (141). The genome-wide associations at multiple chromosomal loci other than 3 and 9 also have been reported, such as on chr12q24.13 (rs10735079) in a gene cluster encoding antiviral restriction enzyme activators (*OAS1*, *OAS2*, *OAS3*), on chr19p13.2 (rs2109069) near the gene encoding *tyrosine kinase 2 (TYK2)*, on chr19p13.3 (rs2109069) within the gene encoding *dipeptidyl peptidase 9 (DPP9)*, and on chr21q22.1 (rs2236757) in the *IFNAR2* gene (103, 140). In contrast, a recent study found that a 75-kb haplotype on chromosome 12 (113,350,796 to 113,425,679 base pairs, rs1156361) associated with a ∼22% reduction in relative risk of developing severe COVID-19 (142). Individuals with human leukocyte antigen (HLA) variants, B*46:01 and B*15:03, respectively, were suggested to bear more and less the susceptibility to SARS-CoV-2 and severity of COVID-19 (143).

The role of epigenetic mechanisms in host immune response in COVID-19 has also been demonstrated by a recent *in vitro* study using genome-editing CRISPR (clustered regularly interspaced short palindromic repeats) screens in Vero-E6 cells. Authors identified epigenetic regulatory molecules High mobility group box 1 (HMGB1) and SWItch/Sucrose Non-Fermentable (SWI/SNF) chromatin remodeling complex—critical for CoV-induced host cell death, including SARS-CoV-2 (144).

People with blood group O were found to have slightly lesser chances of getting COVID-19 as well as relative protection from developing severe symptoms and death in comparison to persons with blood groups A, B, and AB (145–147). Of note, a very recent case-control study involving a very large data of COVID-19 patients (more than 11,000 positive cases) neither found increased risks in persons with A, B, and AB blood groups nor any protection for O blood group for contracting infection, hospitalization, disease severity, and outcomes (148).

Autoimmunity has also been an issue in the pathogenesis of severe symptoms in COVID-19. A study observed that at least 10.2% of the patients who are aged 25–87 years had autoantibodies against IFN type I, of which 95 (94%) were men. A further instance of generation of autoantibodies in COVID-19 patients has been noted against host cell phospholipids (149). The presence of prothrombotic antiphospholipid (aPL) antibodies in the serum of COVID-19 patients could also be a reason for enhanced thrombotic incidences in some patients (150, 151). In contrast, a study by Borghi et al. (152) found a low prevalence of aPL in COVID-19 patients and no association between thrombosis and aPL.

### Gut Microbiome

The gut microbiome is said to have a significant role in disease severity and patient outcomes in COVID-19, as they build up the local mucosal immunity against viral invasions (153). Recent studies showed that patients with COVID-19 have a different microbiome compared with controls, enriched with the opportunistic pathogens, and depletion of the beneficial commensals showed a correlation with severity of the symptoms (65, 154). The studies have further indicated that the composition of the gut microbiome may influence SARS-CoV-2-induced production of inflammatory cytokines and consequently in the onset of a cytokine storm (65, 153, 155).

### Cross Immunity and Protection From Severe Disease

SARS-CoV-2-reactive T cells and antibodies were found present in many individuals without a previous exposure (156, 157), indicating that previous infections with other CoVs might have caused this. Respiratory infections in humans by CoVs, especially those causing common cold are common. Accumulating evidence suggests that existing exposures to common cold strains can be protective from developing severe symptoms, if infected with SARS-CoV-2 (158). Apart from CoVs, infections with other respiratory viruses, recent flu shots (influenza vaccines), and childhood vaccinations with live attenuated bacteria/viruses, such as Bacille Calmette-Guerin (BCG) and measles-mumps-rubella (MMR) may be partially protective (159–162). The exact biological mechanisms for the protection are not known; however, an epigenetic mechanism leading to the “trained immunity” of the myeloid cells from the previous exposures to the related pathogens may be a plausible reason for this, with the information available from the existing literature (163).

## Possible Pathophysiology of “Long COVID”

Persistence or recurrence of the clinical symptoms as “long COVID” has turned up as a significant health issue in the discharged patients of COVID-19 (17–19). In addition to the persistence or recurrence of certain clinical symptoms, a possible risk of infertility in men, chronic fatigue, and the new onset of diabetes in COVID-19 survivors have been reported (17, 19, 72, 164). Furthermore, the formation of autoantibodies in many COVID-19 patients has been observed, which indicates that the survivors may have an increased risk of autoimmune disorders (149, 165). Newly developed disabilities (165, 166), and a likely decrease in life expectancy as being indicated by recent studies (167, 168), are other key concerns in the survivors. Long-term health issues are also noted in infection with other BCoVs, such as SARS-CoV-1 and MERS-CoV (169); however, the reasons have not been well understood yet. At present, its pathophysiology is not clearly understood; however, various speculations are being presented based on the known facts of the virus–host interactions in COVID-19. In general, the chronic illness and subsequent scarring and dysfunction of the affected organs may be the reasons for the long-term health issues (167). Apart from that, the chronic infection with SARS-CoV-2 may induce epigenetic changes in vital organs, thus reprogramming its functions, as have been recently demonstrated in a murine model of COVID-19, which may set the platform for long-term health issues (38). Profound inhibition of cell growth in the virally infected cells across the organs may be another potent molecular mechanism leading to long-term health issues (37). The cell growth inhibition may particularly affect the tissues with a high mitotic rate like reproductive and endocrine organs, mucosal and vascular epithelium, and neurogenic brain regions; hence, health issues, including those related to learning and memory may particularly arise in the long-term in COVID-19 survivors.

## Changing Face of the Pandemic: A Shift in Host–Virus Interactions With Emerging SARS-CoV-2 Variants

**Figure 7:**
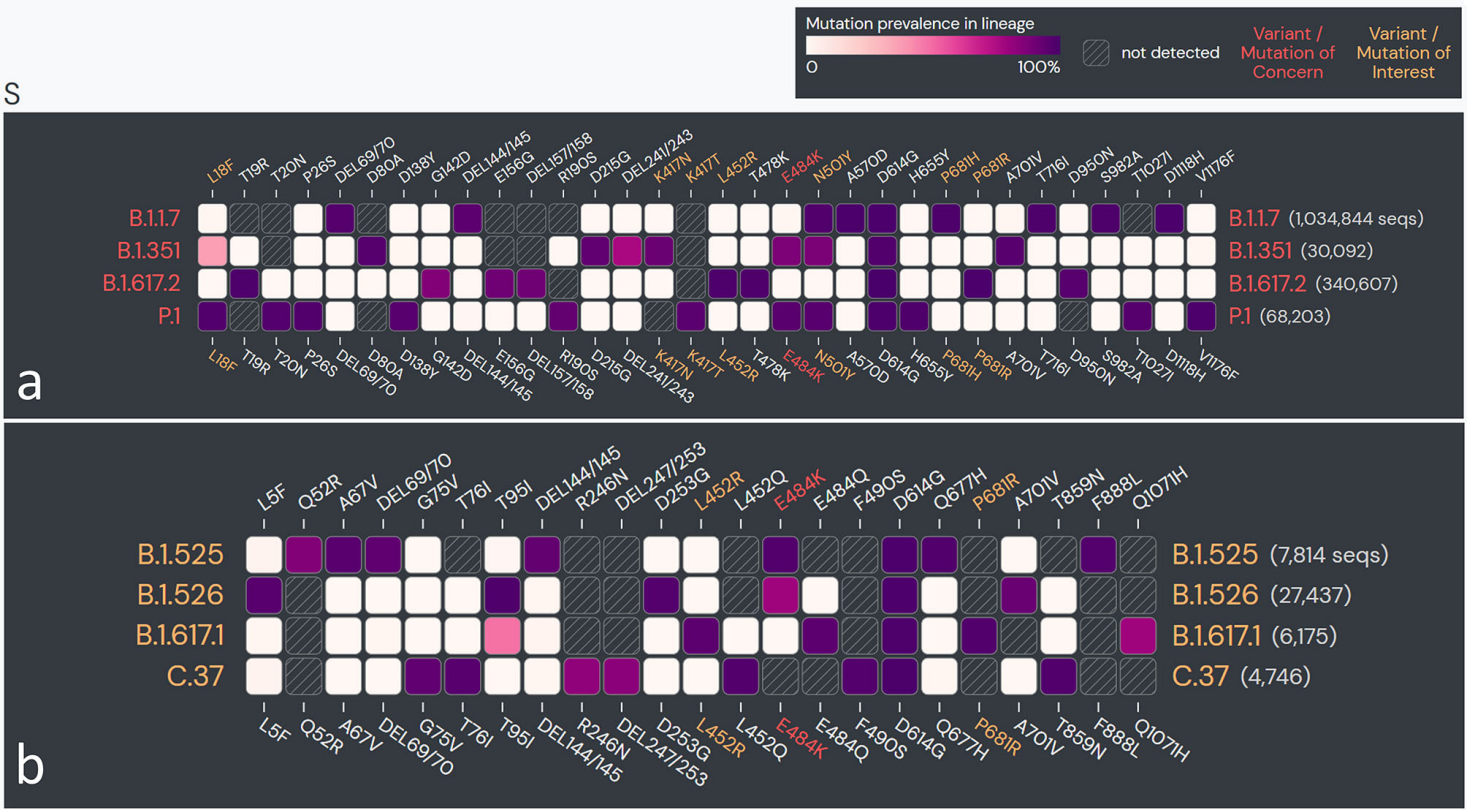
The spike protein coding sequence mutations in emerging severe acute respiratory syndrome coronavirus 2 (SARS-CoV-2) variants globally. **(A)** Variants of concern (VOCs). **(B)** Variants of interest (VOIs). [Analysis included mutations with >75% prevalence in at least one lineage currently recognized by World Health Organization (WHO) as variants of concern and variants of interest globally. Data source: www.outbreak.info, accessed on 28/08/2021].

The vast spread of the first COVID-19 wave beyond geographical boundaries had supposedly created an immunological barrier in the infected population against the wild-type (WT) SARS-CoV-2 strain (nCoV-2019), hopefully limiting recurrent waves. In a paradox, the massive new COVID-19 waves driven by emerging SARS-CoV-2 variants have appeared in the years 2020–2021 across the globe (170–173). The emerging SARS-CoV-2 variants, which seem to have greater transmissibility and virulence and are capable of escape against natural and acquired (from vaccines and therapeutically used monoclonal antibodies) immunity against the WT strains (172–175) (**Table 2**), have doomed the hope of a sooner ending of the pandemic. Recent *in situ* and animal models and clinical studies have confirmed that the variants have a shorter incubation period, higher viral load, and prolonged viral shedding (176–181). The variants may cause greater damage in the infected host tissue; however, if there has been a change in the tissue type/organ-specific pathogenesis is currently least understood (172). WHO has identified four variants of concerns (VOCs) and four variants of interest (VOIs) globally (173). The SARS-CoV-2 variants are characterized by lineage-specific key mutations in the spike protein regions, which are said to contribute to increased transmissibility and/or virulence and immune escape to natural and vaccine-acquired antibodies (3, 172, 176, 177) (**Figure 7**). Many of the spike protein mutations are shared across the variants indicating their convergent evolution and selective advantage for the epidemiological fitness (2) (**Figure 7**). The lineage-specific mutations are also present in the non-spike regions of the variants (182); however, currently, there is little known about their epidemiological significance.

**Table 2:** Emerging severe acute respiratory syndrome coronavirus 2 (SARS-CoV-2) variants across the globe and their clinical–epidemiological characteristics.

WHO label	Pangolineages	Variant status (WHO/CDC)	GISAID clade	Nextstrainclade	Key Spike mutations (frequency >75%)*	First reported	Date of designation	Transmission*	Lethality*	Immunoescape*
***Alpha***	B.1.1.7	Variant of concern (VOC)#	GRY	20I (V1)	69del, 70del, 144del, E484K, S494P, N501Y, A570D, D614G, P681H, T716I, S982A, D1118H K1191N	United Kingdom, Sep-2020	18-Dec-2020	~50% increased transmission compared to B.1	Potential increased severity based on hospitalizations and case fatality rates	• No impact on susceptibility to monoclonal antibody treatments • Minimal impact on neutralization by convalescent and post-vaccination sera
***Beta***	B.1.351 B.1.351.2 B.1.351.3	VOC#	GH/501Y.V2	20H (V2)	69del, 70del, 144del, E484K, S494P, N501Y, A570D, D614G, P681H, T716I, S982A, D1118H K1191N	South Africa, May-2020	18-Dec-2020	~50% increased transmission	More lethal	• Significant decrease in susceptibility to the combination of bamlanivimab and etesevimab monoclonal antibody treatment • Reduced neutralization by convalescent and post-vaccination sera
***Gamma***	P.1 P.1.1 P.1.2 P.1.4 P.1.6 P.1.7	VOC#	GR/501Y.V3	20J (V3)	L18F, T20N, P26S, D138Y, R190S, K417T, E484K, N501Y, D614G, H655Y, T1027I	Brazil, Nov-2020	11-Jan-2021	Not ascertained yet	More lethal	• Significant decrease in susceptibility to the combination of bamlanivimab and etesevimab monoclonal antibody treatment • Reduced neutralization by convalescent and post-vaccination sera
***Delta***	B.1.617.2 AY.1 AY.2 AY.3 AY.3.1	VOC#	G/478K.V1	21A	T19R, (G142D), 156del, 157del, R158G, L452R, T478K, D614G, P681R, D950N	India, Oct-2020	VOI: 4-Apr-2021 VOC: 11-May-2021	~50-60% increased transmission compared to B.1.1.7	Preliminary results suggest 2.61 times higher the risk of hospitalization within 14 days compared with the B.1.1.7.	• Potential reduction in neutralization by some monoclonal antibody treatments • Potential reduction in neutralization by post-vaccination sera
***Lambda***	C.37	Variant of interest (VOI) #	GR/452Q.V1	21G	D614G, L452Q, F490S, T859N, T76I, G75V, del247/253	Peru, Dec-2020	14-Jun-2021	Not ascertained yet	Not ascertained yet	• Not ascertained yet
***Mu***	B.1.621, B.1.621.1	VOI#	GH	21H	Spike: T95I, Y144S, Y145N, R346K, E484K, N501Y, D614G, P681H, D950N	Colombia, Jan-2021	-Sep-2021	31-Aug-2021	Not ascertained yet	• Not ascertained yet
***Eta***	B.1.525	Variant Being Monitored (VOB)*	G/484K.V3	21D	A67V, 69del, 70del, 144del, E484K, D614G, Q677H, F888L	Multiple countries, Dec-2020	17-Mar-2021	Not ascertained yet	Not ascertained yet	• Potential reduction in neutralization by some monoclonal antibody treatments • Potential reduction in neutralization by convalescent and post-vaccination sera
***Iota***	B.1.526	Variant Being Monitored (VOB)*	GH/253G.V1	21F	L5F, T95I, D253G, S477N, E484K, D614G, A701V	United States of America, Nov-2020	24-Mar-2021	Not ascertained yet	Not ascertained yet	• Reduced susceptibility to the combination of bamlanivimab and etesevimab monoclonal antibody treatment. • Reduced neutralization by convalescent and post-vaccination sera
***Kappa***	B.1.617.1	Variant Being Monitored (VOB)*	G/452R.V3	21B	T95I, G142D, E154K, L452R, E484Q, D614G, P681R, Q1071H	India, Oct-2020	4-Apr-2021	More transmissible	Increased lethality in animal model. In humans not ascertained yet	• Potential reduction in neutralization by some monoclonal antibody treatments • Potential reduction in neutralization by post-vaccination sera

^#^Based on latest updates by WHO, Geneva (173). *Based on latest updates by Centers for Disease Control and Prevention (CDC), USA (174).

## Plausible Mechanisms for Enhanced Transmissibility/Virulence and Immune Escape Capabilities in Variants

Increased transmissibility and/or virulence has been reported for nearly all of the VOCs and multiple VOIs (174) (**Table 2**). The spike protein regions bear the binding site for the host cell entry receptor and the natural and acquired host antibodies. Key mutations in the spike protein (**Table 2**), chiefly in the receptor-binding domain (RBD), are believed to induce conformational changes resulting in stronger binding to the key host cell entry receptor ACE2 (2, 3). Moreover, certain mutations have created newer contact sites or stronger electrostatic or newer hydrogen bonds between RBD and host ACE2 (2) [Reviewed in (176, 177)].

Interestingly, the mutations in the spike protein sequence of certain variants (B.1.1.7 and B.1.617 lineage) occurring at amino acid position 681 (P—H/R), which falls in the FCS, improve proteolytic cleavage of the spike protein strengthening fusion of the viral membrane with the host cell membrane [Reviewed in (1)]. An improved viral–host cell fusion may supposedly result in larger syncytia formation (1). Syncytia formation, a fusion of the infected host cell with other cells, is believed to facilitate viral spread, thus imparting higher transmissibility to the variants (183–185). Notably, syncytia formation has been a distinctive characteristic of SARS-CoV-2 when compared to SARS-CoV-1 [Reviewed in (1)].

Other than increased transmissibility and virulence, recent studies have shown that most of the variants, primarily VOCs, have gained a certain level of resistance against the natural and acquired (from vaccines and therapeutically used monoclonal antibodies) immunity (174) (**Table 2**). Frequent repeated and vaccine breakthrough infections have been reported with the variants (172, 186, 187). The exact mechanisms for the gain of immune escape capabilities in variants are currently not well understood. Although, based on the emerging literature, the most likely immune escape mechanisms are (i) inclusion or deletion of amino acid residue at immunogenic epitopes (for the natural and acquired antibodies) bringing conformational changes at the binding interface (2–4, 176, 177); (ii) remodeling of the electrostatic surface potential at the antigen–antibody binding interface (2–4, 176, 177); and (iii) gain of additional glycosylation sites, which shield the binding site for the neutralizing antibodies (188). Future studies are warranted that can unravel the mechanisms that the variants utilize for the immune escape.

## Concluding Remarks

Extensive research has been performed globally, unraveling the various mechanisms involved in the pathogenesis and host immune responses for COVID-19. These studies have mainly targeted viral proteomics and genomics and host-dependent factors. Extensive experimental studies have been conducted involving both cell culture-based and animal models of COVID-19. Furthermore, a human-specific adaptation of the viral–host interaction mechanisms has been derived from the laboratory data from the patients with COVID-19 and the experimental studies involving human tissue organoids. In light of the extensive findings achieved so far, we currently have a broad understanding of the virus–host interactions, tissue tropism and organ-specific pathogenesis, involvement of physiological systems, and the human immune response against the SARS-CoV-2 infection. The widespread expression of SARS-CoV-2 host cell entry factors across human tissue types, RAAS dysregulation, and a hyperactive innate immune response accompanied by delayed or suppressed adaptive immunity seem to be key factors behind systemic manifestations and poor clinical outcomes in the patients. The inclusion of FCS in the spike protein sequence may be a reason for the increased virulence of SARS-CoV-2. Additionally, dampening of the early IFN response and subsequent cytokine storm and suppressed/delayed adaptive immune response marked by intense lymphocytopenia and suboptimum synthesis of immunoglobulins are the prominent immunological features, which seem to drive the severity of the disease. The preexisting genetic factors may be the prime reason behind the increased vulnerability of certain individuals for contracting the infection and the severity of the disease; however, little is known on this issue yet. Gut microbiome may also have a significant role in disease outcomes as has been indicated from the emerging literature. Multiple knowledge gaps in aspects of the disease are remaining, which need to be addressed in future studies. Furthermore, the persistence or recurrence of the symptoms as “long COVID” is a very important health concern that needs to be intensively researched. Most importantly, the emerging SARS-CoV-2 variants driving recurrent COVID-19 waves, imparted by increased transmissibility/virulence and immune escape capabilities, requires further in-depth research to address its mechanism(s) precisely.

## Author Contributions

AK wrote the first draft. GK, SaK, CK, MK, PP, RN, VP, MF, PS, KS, KK, SP, HS, and SuK revised the draft. RN performed data analysis, and PP and RN prepared the figures. All authors contributed to the article and approved the submitted version.

## Conflict of Interest

The authors declare that the research was conducted in the absence of any commercial or financial relationships that could be construed as a potential conflict of interest.

## Publisher’s Note

All claims expressed in this article are solely those of the authors and do not necessarily represent those of their affiliated organizations, or those of the publisher, the editors and the reviewers. Any product that may be evaluated in this article, or claim that may be made by its manufacturer, is not guaranteed or endorsed by the publisher.
